# In-Situ Simulation in Interdisciplinary Family Practice Improves Response to In-Office Emergencies

**DOI:** 10.7759/cureus.14315

**Published:** 2021-04-06

**Authors:** Brad D Gable, Laurie Hommema

**Affiliations:** 1 System Medical Director, Simulation, Riverside Methodist Hospital, Columbus, USA; 2 Medical Director, Provider and Associate Well-Being, Ohio Health Associate Program Director, Riverside Family Medicine Residency, OhioHealth, Columbus, USA

**Keywords:** simulation medicine, skills and simulation training, interdisciplinary simulation, outpatient family medicine, emergency, in-situ simulation

## Abstract

Background

Medical emergencies can present to family medicine offices. For optimal patient outcomes, multiple team members must come together to provide emergency care and mobilize the appropriate resources. In-situ simulation has been used to improve provider knowledge, skills, and attitudes as well as identify latent safety threats. The aim of this training was to provide family medicine physicians, nurses, and office staff education about how to manage in-office emergencies. Specifically, we sought to clarify team members’ roles, improve communication, and identify latent safety threats.

Methodology

Two different in-situ simulations were performed with debriefing sessions. The first was a pediatric patient in respiratory distress. The second was a patient who presented for shortness of breath and became unresponsive in the lobby. Physicians, nurses, and office staff responded to the emergencies and used existing equipment and protocols to medically manage each patient. A standardized return on investment in learning survey evaluating the learners’ confidence in managing in-office emergencies was completed by all learners immediately prior to and after the training.

Results

The training improved the participants’ self-reported confidence in their ability to manage in-office emergencies. Additionally, participants believed they were better able to identify other team members’ roles when responding to an in-office emergency. Learners were able to identify where knowledge gaps existed in current protocols, as well as aspects of the protocols that required updating. Lastly, the teams identified latent safety threats that were able to be mitigated by the practice.

Conclusions

In-situ simulation for high-risk, low-frequency in-office emergencies is a valuable tool to improve team members’ confidence, identify knowledge gaps, and mitigate latent safety threats.

## Introduction

Simulation in healthcare can be defined as a “tool, device, and/or environment (that) mimics an aspect of clinical care” [1]. Simulation has been valuable in healthcare to demonstrate areas for improvement within systems and processes, as well as to evaluate new approaches prior to deployment in actual clinical environments [2,3]. Specifically, in-situ simulation has previously been used to identify latent safety threats [4]. In-situ simulation has inherent challenges such as relocating simulation equipment and potentially disrupting patients and staff [4,5].

In-office medical emergencies in primary care are rare [6]. Time is of the essence during these situations and early cardiopulmonary resuscitation and defibrillation have been shown to improve outcomes during out-of-hospital cardiac arrest [7]. Only recently have any studies sought to determine the impact of simulation training on primary care office staff confidence in managing these emergencies [8,9]. The aim of this training was to provide family medicine physicians, nurses, and office staff education about how to manage in-office emergencies. Specifically, we sought to clarify team members’ roles, improve communication, and identify latent safety threats.

## Materials and methods

Our simulation training occurred during protected time for our family medicine residents. The primary care office was closed to patients during this time. Learners included clinical staff (attending physicians, residents, medical students, registered nurses, and medical assistants) as well as non-clinical staff (office specialists). Participants rotated through two simulations, each lasting one hour (Table 1). Simulation 1 was a pediatric patient in respiratory distress in an exam room. Simulation 2 was an unresponsive patient in the lobby bathroom.

**Table 1:** In-situ simulation cases.

Simulation	Type of education	Equipment	Location	Learners	Goals	Case
1 – Pediatric respiratory distress patient	In-situ simulation	High-fidelity mannequin standardized patient	Exam room	Attendings, residents, medical students, nurses, medical assistants, office staff	Use current equipment and protocols to evaluate, treat, and disposition a pediatric patient in respiratory distress	8-year-old male with a history of asthma presented with grandfather. Has been trying to use inhaler because of increasing wheezing but is getting worse
2 – Unresponsive patient	In-situ simulation	High-fidelity mannequin	Lobby	Attendings, residents, medical students, nurses, medical assistants, office staff	Use current equipment and protocols to evaluate, treat, and disposition an unresponsive patient	Middle-aged male presented with his wife for evaluation of worsening shortness of breath. While waiting in the lobby became unresponsive, apneic, and pulseless

Surveys of the learners were conducted in a pre-post format. Participants were asked about their confidence in their ability to respond to unresponsive patients and pediatric patients in respiratory distress. Additionally, they were asked if they understood all team members’ roles when responding to these emergencies. Additional data regarding the educational event were asked post-survey. Data analysis was performed in aggregate and reported as means. Matched data were further analyzed with a paired t-test, and statistical significance was evaluated with p-values using a two-tailed test. This project was deemed to be not human subjects research by the OhioHealth Institutional Review Board, and is therefore exempt.

The evaluation and measurement strategy employed the use of the return on investment (ROI) methodology for program evaluation [10]. The data collection plan created to evaluate this education utilized the ROI methodology levels zero through two of evaluation. The data collection plan comprised the following: Level zero - Inputs: Number of learners delineated by the job title. Level one - Reaction: A mean target score of 4 or greater (agree and strongly agree) on the Likert scale-based questions, as scored by the participants on the post-education evaluation. Level two - Learning: A mean target score of 4 or greater (agree and strongly agree) on the Likert scale-based questions, as scored by the participants on the post-education evaluation constituted a successful education event [10].

Additionally, during the debriefing, specific feedback from the learners was captured regarding latent safety threats and knowledge gaps. This information was then given to the program director and practice medical director who determined means for mitigating these threats and knowledge gaps.

## Results

Using the ROI methodology we obtained the following: Level zero - Inputs: A total of 19 learners participated in the education. These included nine resident physicians, three nurses, two medical assistants, three medical students, and two attending physicians. Levels one and two - Reaction and Learning: matched pre and post-survey data were obtained from 17 learners. On the post-survey, 100% of the learners agreed or strongly agreed that they could respond to unresponsive patients and patients in respiratory distress, which was a statistically significant improvement from the pre-education surveys (Table 2). In addition, all learners could identify every team member’s role in these emergencies. The training was well liked by the learners (Table 3). More than 90% of the learners felt the training was relevant and planned to use what they had learned.

**Table 2:** Pre-survey and post-survey confidence data.

Survey questions	Strongly disagree (number responses pre/number responses post)	Disagree (number responses pre/number responses post)	Neutral (number responses pre/number responses post)	Agree (number responses pre/number responses post)	Strongly Agree (number responses pre/number responses post)	Pre-survey confidence mean (95% confidence interval), 1-5 scale	Post-survey confidence mean (95% confidence interval), 1-5 scale	P-Value (significant <0.05)
Q1. I feel confident in my ability to respond to an unresponsive patient in the office	0/0	2/0	3/0	10/7	4/10	4.1 (3.6-4.6)	4.6 (4.4-4.9)	0.014
Q2. I understand team members’ roles when responding to an unresponsive patient in the office	1/0	2/0	4/0	9/8	3/9	3.8 (3.1-4.4)	4.6 (4.3-4.9)	0.006
Q3. I feel confident in my ability to respond to a patient in respiratory distress in the office	0/0	2/0	3/0	11/9	3/8	3.9 (3.4-4.4)	4.5 (4.2-4.8)	0.013
Q4. I understand team members’ roles when responding to a patient in respiratory distress in the office	0/0	1/0	6/0	10/8	2/9	3.7 (3.2-4.2)	4.6 (4.3-4.9)	0.003

**Table 3:** Post-simulation evaluation of the overall education.

Question	Mean (95% confidence interval), 1-5 scale
Q6. This training was relevant to my work	4.69 (4.4-5.0)
Q7. This training provided me with new information (or clarified old information)	4.77 (4.5-5.0)
Q8. I intend to use what I learned from this training	4.77 (4.5-5.0)
Q9. This training would be of benefit to my colleagues	4.85 (4.6-5.0)
Q10. Overall, I thought the training was good/very good	4.69 (4.4-5.0)
Q11. I thought the instructor(s) were good/very good	4.69 (4.4-5.0)

Finally, feedback was captured during the simulations’ debriefings. This information was identified as either a latent safety threat or an educational opportunity (Table 4). The latent safety threats and educational opportunities were provided as feedback to the program director and practice medical director and practice administrator. The following educational improvements were made: (1) Defined process of who calls 911, as well as the institution-specific protocols that must be followed to do so. (2) Defined roles for specific emergencies. (3) Delineation of how to give, execute, and document verbal orders in the office setting. (4) Education around cardiopulmonary resuscitation, bag-valve-mask ventilation, and defibrillation in the office. This is in addition to the institution required Basic Life Support for medical assistants, and Advanced Cardiovascular Life Support for nurses and residents. The following latent safety threats were mitigated: (1) Locked equipment process was changed to improve ease of access while still remaining compliant with Joint Commission regulations; (2) naloxone made available; (3) Mucosal Atomization Device® device made available; (4) glucose gel made available.

**Table 4:** Latent safety threats and educational opportunities identified, as well as mitigation strategies. MAD® = Mucosal Atomization Device; CPR = cardiopulmonary resuscitation

Unresponsive patient case	Pediatric respiratory distress case
Latent safety threats	Latent safety threats
Lack of availability of naloxone or MAD®; lack of glucose gel availability; and lack of emergency response bag (or necessary supplies being within close proximity to one another)	Closet door for respiratory equipment locked; door for portable oxygen locked; need for an emergency response bag
Educational opportunities	Educational opportunities
Compression-only CPR for adult out-of-hospital cardiac arrest; role clarity for calling 911; 911 calls routed through institution’s protective services first and then to local 911 dispatch	Role clarity for who calls 911; how to give, receive, and document verbal medication orders in the office setting; role clarity for what a medical student can do in an office emergency

## Discussion

In-situ simulation is an effective means of providing education about low-frequency, high-risk events. The sterile surroundings of a simulation lab can deliver much of the same information, but lacks the ability to fully replicate the clinical environment in which healthcare providers interact daily. Here, we have demonstrated that in-situ simulation is an effective means of education for management of in-office emergencies. This type of simulation-based education in the in-situ environment provides a means of not only delivering new educational content and skills but also identifying barriers and enablers to implementation of those skills. After this in-situ simulation education, all participants felt confident in their ability to respond to unresponsive patients and patients in respiratory distress. Additionally, all learners understood their roles, and each team member’s role in responding to in-office emergencies. As a result of this education, we were able to identify and mitigate several latent safety threats. Additionally, we identified knowledge gaps and provide additional information and training. These findings demonstrate that moving away from traditional lecture-based education and toward more experiential learning not only allows learners to gain knowledge and confidence but also to identify and mitigate latent safety threats. This education was performed at a single community-based family medicine residency office and may not be generalizable to other practices. Additionally, our relatively small number of learners allowed for all learners to participate, but this small sample size also limits generalizability. To replicate this in-situ simulation education at larger practices additional resources would be necessary. Further studies on patient-centered outcomes such as morbidity and mortality as a result of in-situ simulation would be of benefit, as would the ROI of an in-office, in-situ simulation program.

## Conclusions

The ability for all members of the healthcare team to perform multi-disciplinary, simulation-based education in the in-situ environment leads to identification and mitigation of latent safety threats, while fostering improved teamwork, communication, and crisis resource management.

## References

[1] Cook DA, Hatala R, Brydges R (2011). Technology-enhanced simulation for health professions education: a systematic review and meta-analysis. JAMA.

[2] Cheng A, Grant V, Auerbach M (2015). Using simulation to improve patient safety: dawn of a new era. JAMA Pediatr.

[3] Slakey DP, Simms ER, Rennie KV, Garstka ME, Korndorffer JR Jr (2014). Using simulation to improve root cause analysis of adverse surgical outcomes. Int J Qual Health Care.

[4] Patterson MD, Geis GL, Falcone RA, LeMaster T, Wears RL (2013). In situ simulation: detection of safety threats and teamwork training in a high risk emergency department. BMJ Qual Saf.

[5] Patterson MD, Blike GT, Nadkarni VM (2008). In situ simulation: challenges and  results. Advances in Patient Safety: New Directions and Alternative Approaches. Vol 3: Performance and Tools.

[6] Liddy C, Dreise H, Gaboury I (2009). Frequency of in-office emergencies in primary care. Can Fam Physician.

[7] Kragholm K, Wissenberg M, Mortensen RN (2017). Bystander efforts and 1-year outcomes in out-of-hospital cardiac arrest. N Engl J Med.

[8] Forde E, Bromilow J, Wedderburn C (2018). Practical management of emergencies in primary care: taking simulation out of the classroom and into real-life environments. BMJ Simul Technol Enhanc Learn.

[9] Lamb EI, Jenkins N, Male P, McFetrich J, Towart M, Sudlow M (2019). Primary care emergencies: improved confidence in clinical and non-clinical members of the multidisciplinary team using a simulation programme. BMJ Simul Technol Enhanc Learn.

[10] Buzachero V, Phillips J, Phillips P, Phillips Z (2013). Measuring ROI in healthcare.

